# Malaria misdiagnosis in Uganda – implications for policy change

**DOI:** 10.1186/1475-2875-8-66

**Published:** 2009-04-16

**Authors:** Joan Nankabirwa, Dejan Zurovac, Julius N Njogu, John B Rwakimari, Helen Counihan, Robert W Snow, James K Tibenderana

**Affiliations:** 1 Malaria Consortium, Africa Regional Office, Sturrock Road, Kampala, Uganda; 2 Malaria Public Health and Epidemiology Group, KEMRI/Wellcome Trust Research Programme, PO Box 43640, 00100 GPO, Nairobi, Kenya; 3 Centre for Tropical Medicine, Nuffield Department of Clinical Medicine, University of Oxford, CCVTM, Oxford, UK; 4 Center for International Health and Development, Boston University School of Public Health, Crosstown 3rd floor, 801 Massachusetts Ave, Boston, MA 02118, USA; 5 Ministry of Health, National Malaria Control Programme, Kampala, Uganda; 6 Malaria Consortium, Development House, 56-64 Leonard Street, London EC2A 4LT; 7 London School of Hygiene and Tropical Medicine, Keppel Street, WC1E 7HT, UK

## Abstract

**Background:**

In Uganda, like in many other countries traditionally viewed as harbouring very high malaria transmission, the norm has been to recommend that febrile episodes are diagnosed as malaria. In this study, the policy implications of such recommendations are revisited.

**Methods:**

A cross-sectional survey was undertaken at outpatient departments of all health facilities in four Ugandan districts. The routine diagnostic practices were assessed for all patients during exit interviews and a research slide was obtained for later reading. Primary outcome measures were the accuracy of national recommendations and routine malaria diagnosis in comparison with the study definition of malaria (any parasitaemia on expert slide examination in patient with fever) stratified by age and intensity of malaria transmission. Secondary outcome measures were the use, interpretation and accuracy of routine malaria microscopy.

**Results:**

1,763 consultations undertaken by 233 health workers at 188 facilities were evaluated. The prevalence of malaria was 24.2% and ranged between 13.9% in patients ≥5 years in medium-to-high transmission areas to 50.5% for children <5 years in very high transmission areas. Overall, the sensitivity and negative predictive value (NPV) of routine malaria diagnosis were high (89.7% and 91.6% respectively) while the specificity and positive predictive value (PPV) were low (35.6% and 30.8% respectively). However, malaria was under-diagnosed in 39.9% of children less than five years of age in the very high transmission area. At 48 facilities with functional microscopy, the use of malaria slide examination was low (34.5%) without significant differences between age groups, or between patients for whom microscopy is recommended or not. 96.2% of patients with a routine positive slide result were treated for malaria but also 47.6% with a negative result.

**Conclusion:**

Current recommendations and associated clinical practices result in massive laria over-diagnosis across all age groups and transmission areas in Uganda. Yet, under-diagnosis is also common in children <5 years. The potential benefits of malaria microscopy are not realized. To address malaria misdiagnosis, Uganda's policy shift from presumptive to parasitological diagnosis should encompass introduction of malaria rapid diagnostic tests and substantial strengthening of malaria microscopy.

## Background

Malaria remains the major public health problem in Uganda with annual estimates of 10 million cases and 43,000 deaths, of which 91% are in children below 5 years of age [1]. Malaria transmission has been traditionally described as stable across 95% of the country, with approximately two thirds of this area classified as very high transmission and the rest as medium-to-high transmission [2,3]. Given this epidemiological context, and the absence of universal coverage of diagnostic services at the periphery of the health system, presumptive diagnosis of febrile episodes as malaria and effective treatment has been the cornerstone of malaria case-management in Uganda [4].

National malaria diagnosis recommendations for health workers are outlined in the recent malaria case-management guidelines and job aids [4-6], which were revised in 2006 during the implementation of a new malaria treatment policy using artemisinin-based combination therapy (ACT), as the first line anti-malarial. According to these guidelines any patient presenting with fever or history of fever in absence of danger signs and prior, correct use of ACT should be presumptively diagnosed as malaria. The use of malaria microscopy is discouraged for most febrile patients with the exception of special patient groups which include suspected treatment failures, severe cases, children under 4 months (or below 5 kg) and pregnant women.

The malaria diagnostic policy in Uganda is currently under revision and a more significant role for parasitological diagnosis is being considered. In support of the policy revision process, this study reports on the accuracy of the current national malaria diagnosis recommendation, the accuracy of routine malaria diagnosis practices, and evaluates the use, interpretation and accuracy of malaria microscopy at facilities where this diagnostic service is available.

## Methods

### Study design and data collection

Between 29 May and 16 August 2007, outpatient malaria case-management was evaluated at all government and private not-for-profit health facilities in four Ugandan districts (Mubende, Jinja, Tororo and Apac). The survey methods are described in detail elsewhere [7]. Briefly, the survey was a cross-sectional, cluster sample survey undertaken at all facilities in four study districts. Data at each facility were collected over one day using quality-of-care assessment methods including health facility assessments, health worker interviews, and exit interviews with all caretakers of sick children and adult outpatients seen during the survey days. Patients coming for follow up visits for chronic conditions (e.g. TB, diabetes, traumas, burns) and patients referred or admitted for hospitalization were not recruited. During the exit interview, study nurses collected information about the patient's age, weight, temperature, history of fever, pregnancy status, main complaints, prior use of anti-malarial drugs, and if the visit was an initial or follow-up consultation. Information was also collected from patient-held records about routine diagnostic procedures requested, results reported, and medications prescribed.

In addition, all patients had, on exit, a finger-prick blood sample taken for malaria microscopy by study laboratory technicians. Thick and thin blood slides were prepared at the facility and stained with 10% Giemsa. At post-survey, two expert microscopists examined independently all study slides at a laboratory based at Malaria Consortium in Kampala. For each study slide 100 high-power magnification fields were examined before the slide was reported as negative. Where results between the two expert microscopists were discordant, a third microscopist re-read the blood slide and the majority decision was accepted as the final result.

### Definitions and statistical analysis

The primary analysis focused on 1) the accuracy of fever as the national malaria diagnosis recommendation, and 2) the accuracy of routine malaria diagnosis practices. To reflect national guideline recommendations for presumptive malaria diagnosis, the primary analysis was restricted to non-pregnant patients weighing ≥5 kg, without prior use of effective anti-malarials, and presenting to the health facility for an initial, outpatient visit. Fever was defined as axillary temperature of ≥37.5°C and/or the history of fever during the present illness. Routine malaria diagnosis was defined as a patient either diagnosed for malaria or treated with an anti-malarial drug. This adjustment was done since 8% of patients did not have any diagnosis written in their cards but all of them had an anti-malarial prescribed and, therefore, it was obvious that a malaria diagnosis was considered.

The secondary analysis evaluated the use, interpretation and accuracy of routine malaria microscopy at health facilities where this diagnostic service was functional during the survey day. The analysis on the use and interpretation of routine malaria microscopy included all patients at these facilities. The clinical process was stratified for patients with and without fever, and subsequently patients with fever were stratified into those for whom the guidelines discourage use of microscopy (i.e. recommend presumptive diagnosis) and those for whom microscopy is recommended (patients weighing <5 kg, pregnant women, and those coming for follow up visit or having prior use of effective anti-malarials). For those patients who had a routine malaria slide performed, further stratification was done by the result of the routine malaria test and the anti-malarial treatment prescribed.

The sensitivity, specificity, positive predictive values (PPVs) and negative predictive values (NPVs) of the policy recommendations and current malaria diagnostic practices were calculated, using standard formulae, against the study malaria case definition, i.e. the presence of any parasitaemia in patients with fever after expert slide examination. For those patients who had a routine malaria slide performed, the accuracy of the routine slide result was evaluated using the same accuracy measures against the presence of any parasitaemia on expert slide examination. In all analyses, the precision of proportions (95% confidence interval [CI]) was determined adjusting for the cluster sampling. All analyses were performed across all age groups and separately for patients below and ≥5 years of age. The small sample size precluded meaningful comparisons between individual districts, thus the results are presented from all districts and stratified by the two districts of medium-to-high transmission (Mubende and Jinja: entomological inoculation rates 4 and 6 respectively) versus the two districts of very high transmission (Tororo and Apac: entomological inoculation rates 562 and 1,586 respectively) [2].

All data were double-entered into Microsoft Access 2000 by two independent data entry clerks and data files were compared for errors by using a verification programme and referring to original data collection forms. All analyses were performed using STATA, version 9.2 (StataCorp, College Station, Texas).

### Ethical approval

Ethical approval for this study was provided by the Uganda National Council for Science and Technology (reference number HS 275).

## Results

### Sample description

In total, 1,763 outpatient consultations undertaken by 233 health workers at 188 health facilities were evaluated. Of 1,763 patients, 278 patients were excluded from the primary analysis evaluating accuracy of malaria diagnosis recommendations and routine malaria diagnosis because they either did not meet the inclusion criterion (n = 274) or had missing results for expert slide examination (n = 4). Therefore, this analysis included 1,485 patients, of which 494 (33.3%) were children below five years of age and 991 (66.7%) were patients five years and older. Of 1,485 patients, 907 were evaluated in the medium-to-high transmission intensity districts (Mubende and Jinja) and 578 in the very high transmission intensity districts (Tororo and Apac). Children below five years of age comprised 288 (31.8%) patients in the medium-to-high and 206 (35.6%) in the very high transmission intensity districts.

For the secondary analysis, the routine clinical process was evaluated from 473 consultations undertaken by 70 health workers at 48 health facilities with functional microscopy on the survey day (26% of all facilities). There were 149 (31.5%) observations for children below five years of age and 324 (68.5%) for patients five years and older. Among all patients 163 routine malaria slides were performed, for which five (3%) expert slide results were not available. Therefore, the accuracy of the routine malaria microscopy was evaluated on 158 slides, of which 48 (30.4%) were for children below five years of age and 110 (69.6%) were for patients ≥5 years of age.

### Background prevalence of key parameters in the study

Table 1 presents results on the background prevalence of malaria parasitaemia, malaria disease, fever, and routine malaria diagnosis stratified by age and malaria transmission area. Of the 1,485 patients meeting our inclusion criteria for primary analysis (non-pregnant patients, weighing ≥5 kg, without prior use of effective anti-malarials, and presenting for an initial, outpatient visit), the overall prevalence of malaria parasitaemia present by expert microscopy was 27.8%. Overall and across both transmission settings, children below five years of age were more commonly parasitaemic than patients five years and older. The prevalence of malaria parasitaemia ranged from 16.8% in patients ≥5 years of age in medium-to-high transmission area to as high as 53.9% in children below five years of age in very high transmission area. The prevalence of malaria disease according to the study definition (presence of any parasitaemia by expert slide examination in a patient with fever) was only marginally lower than any parasitaemia (overall 24.2%) and ranged across all categories from 13.9% in patients five years and older in medium-to-high transmission areas to 50.5% for children in very high transmission areas (Table 1).

**Table 1:** Background prevalence of malaria parasitaemia, malaria disease, fever, and routine malaria diagnosis, stratified by transmission intensity and age categories

	**Very high transmission districts** **n,% (95% CI)**	**Moderate-to high transmission districts** **n,% (95% CI)**	**All districts** **n,% (95% CI)**
**All age groups**	N = 578	N = 907	N = 1485

Malaria parasitaemia	203, 35.1 (31.0–39.3)	210, 23.2 (19.9–26.4)	413, 27.8 (25.0–30.6)

Malaria disease*	175, 30.3 (26.3–34.3)	184, 20.3 (17.1–23.5)	359, 24.2 (21.5–26.8)

Fever prevalence	477, 82.5 (78.6–86.5)	699, 77.1 (73.6–80.5)	1176, 79.2 (76.6–81.8)

Routine malaria diagnosis	423, 73.2 (68.1–78.3)	624, 68.8 (64.0–73.6)	1047, 70.5 (67.0–74.1)

**Patients <5 years**	N = 206	N = 288	N = 494

Malaria parasitaemia	111, 53.9 (48.6–59.2)	106, 36.8 (29.6–44.0)	217, 43.9 (39.0–48.9)

Malaria disease*	104, 50.5 (44.9–56.1)	98, 34.0 (26.8–41.2)	202, 40.9 (35.9–45.9)

Fever prevalence	190, 92.2 (88.2–96.3)	248, 86.1 (82.2–90.0)	438, 88.7 (85.8–91.5)

Routine malaria diagnosis	170, 82.5 (76.3–88.8)	218, 75.7 (69.8–81.6)	388, 78.5 (74.2–82.8)

**Patients ≥5 years**	N = 372	N = 619	N = 991

Malaria parasitaemia	92, 24.7 (20.1–29.3)	104, 16.8 (13.7–19.9)	196, 19.8 (17.1–22.4)

Malaria disease*	71, 19.1 (15.2–23.0)	86, 13.9 (10.9–16.9)	157, 15.8 (13.5–18.2)

Fever prevalence	287, 77.2 (71.6–82.7)	451, 72.9 (68.3–77.4)	738, 74.5 (71.0–78.0)

Routine malaria diagnosis	253, 68.0 (61.8–74.2)	406, 65.6 (60.0–71.2)	659, 66.5 (62.3–70.7)

* Defined as any malaria parasitaemia on expert slide examination in patient with fever

Conversely, the overall prevalence of fever was high (79.2%) and was significantly higher in children under five years (88.7%) than in patients five years and older (74.5%). The higher prevalence of fever was observed in very high transmission areas (82.5%) compared to medium-to-high transmission areas (77.1%), however the difference was not statistically significant (Table 1). Equally, routine malaria diagnosis was commonly made and largely mirrored the age and transmission patterns of fever (Table 1). Overall, of the 1,485 patients evaluated, 70.5% were diagnosed for malaria, more frequently in children below five years of age (78.5%) compared to patients five years and older (66.5%). No statistically significant difference was observed between medium-to-high and very high malaria transmission areas, neither across all age groups (73.2% vs 68.8%) nor within particular age categories (82.5% vs 75.7% in children below five and 68.0% vs 65.6% in patients ≥5 years of age).

### Accuracy of policy recommendations and routine malaria diagnosis

The malaria case-management policy recommending presumptive diagnosis of fever as malaria had high sensitivity and NPV (by our definition 100%), however, the specificity and PPV of these recommendations were very low (27.4% and 30.5% respectively) across both age groups and transmission settings (Table 2). The magnitude of malaria over-diagnosis (1-PPV) differed between transmission settings and age groups, ranging from the lowest, 45.3% in children below five years of age in very high transmission area, to as high as 80.9% in patients ≥5 years of age in medium-to-high transmission area.

**Table 2:** Specificity and positive predictive value (PPV) of fever as policy recommendations compared with malaria disease*, stratified by transmission intensity and age categories

	**Very high transmission districts** **n/N,% (95% CI)**	**Moderate-to-high transmission districts** **n/N,% (95% CI)**	**All districts** **n/N,% (95% CI)**
**All age groups**			

Specificity	101/403, 25.1 (19.7–30.5)	208/723, 28.8 (24.7–32.9)	309/1126, 27.4 (24.2–30.7)

PPV	175/477, 36.7 (32.1–41.3)	184/699, 26.3 (22.4–30.3)	359/1176, 30.5 (27.4–33.6)

**Patients <5 years**			

Specificity	16/102, 15.7 (7.9–23.4)	40/190, 21.1 (15.8–26.3)	56/292, 19.2 (14.9–23.5)

PPV	104/190, 54.7 (49.1–60.4)	98/248, 39.5 (31.8–47.3)	202/438, 46.1 (41.0–51.3)

**Patients ≥5 years**			

Specificity	85/301, 28.2 (21.7–34.7)	168/533, 31.5 (26.4–36.6)	253/834, 30.3 (26.4–34.3)

PPV	71/287, 24.7 (20.0–29.5)	86/451, 19.1 (15.1–23.0)	157/738, 21.3 (18.2–24.3)

* Defined as any malaria parasitaemia on expert slide examination in patient with fever

In comparison to the accuracy of national case-management recommendations, routine malaria diagnostic practices of health workers resulted in somewhat higher specificity (35.6% vs 27.4%) and lower sensitivity (89.7% vs 100%) (Table 3). The over-diagnosis rates (1-PPV) of routine malaria diagnosis were similar to those of national recommendations and they ranged from 47.1% to 80.5% across all categories. Importantly, routine malaria diagnostic practices had higher rates of malaria under-diagnosis (1-NPV) compared to national recommendations and they ranged from the lowest 3.3% in patients = 5 years of age in medium-to-high transmission area to as high as 39.9% in children below five years of age in very high transmission area (Table 3).

**Table 3:** Accuracy of routine malaria diagnosis compared with malaria disease*, stratified by transmission intensity and age categories

	**Very high transmission districts** **n/N,% (95% CI)**	**Moderate-to-high transmission districts** **n/N,% (95% CI)**	**All districts** **n/N,% (95% CI)**
**All age groups**			

Sensitivity	150/175, 85.7 (79.6–91.8)	172/184, 93.5 (89.9–97.1)	322/359, 89.7 (86.1–93.3)

Specificity	130/403, 32.3 (26.4–38.1)	271/723, 37.5 (32.1–42.9)	401/1126, 35.6 (31.6–39.6)

PPV	150/423, 35.5 (30.8–40.1)	172/624, 27.6 (23.4–31.8)	322/1047, 30.8 (27.6–34.0)

NPV	130/155, 83.9 (77.8–90.0)	271/283, 95.8 (93.6–97.9)	401/438, 91.6 (88.7–94.4)

**Patients <5 years**			

Sensitivity	90/104, 86.5 (79.7–93.4)	93/98, 94.9 (90.6–99.2)	183/202, 90.6 (86.4–94.8)

Specificity	22/102, 21.6 (12.6–30.5)	65/190, 34.2 (26.7–41.7)	87/292, 29.8 (24.0–35.6)

PPV	90/170, 52.9 (46.6–59.3)	93/218, 42.7 (34.4–50.9)	183/388, 47.2 (41.8–52.6)

NPV	22/36, 61.1 (45.8–76.4)	65/70, 92.9 (86.9–98.8)	87/106, 82.1 (74.5–89.7)

**Patients ≥5 years**			

Sensitivity	60/71, 84.5 (75.2–93.9)	79/86, 91.9 (86.1–97.6)	139/157, 88.5 (83.3–93.7)

Specificity	108/301, 35.9 (29.3–42.4)	206/533, 38.7 (32.7–44.6)	314/834, 37.7 (33.2–42.1)

PPV	60/253, 23.7 (18.4–29.0)	79/406, 19.5 (15.4–23.5)	139/659, 21.1 (17.9–24.3)

NPV	108/119, 90.8 (85.8–95.7)	206/213, 96.7 (94.5–99.0)	314/332, 94.6 (92.2–96.9)

* Defined as any malaria parasitaemia on expert slide examination in patient with fever

### Use, interpretation and accuracy of routine malaria microscopy

Of the 473 patients at facilities with functional microscopy, 342 (72.3%) presented with fever and 131 (27.7%) without fever. Among the patients with fever, there were 57 for whom the national guidelines recommend the use of microscopy, while the remaining 285 febrile patients were cases for whom malaria microscopy is discouraged. Among all patients (473), the overall use of routine malaria microscopy was low (34.5%; 95% CI: 23.4–45.6) and there were no differences in microscopy use between children below five years of age (50/149; 33.6%; 95% CI: 21.4–45.7) and patients five years and older (113/324; 34.9%; 95% CI: 23.0–46.8). Patients for whom guidelines recommend the use of microscopy were more frequently routinely tested (45.6%; 95% CI: 28.4–62.9) than the patients with fever for whom microscopy is discouraged (39.5%; 95% CI: 27.4–51.5) and patients without fever (28.4%; 95% CI: 9.8–33.0), however the differences between these categories of patients were not statistically significant.

Overall, there were 79 positive and 84 negative slides routinely reported. Nearly all patients with a positive slide result were treated for malaria (96.2%; 95% CI: 91.8–100). Yet, health workers prescribed an anti-malarial treatment for 47.6% (95% CI: 31.3–64.0) of patients with a routinely reported negative slide result. Conversely, health workers respected a negative test result and did not prescribe an anti-malarial for 52.4% (95% CI: 36.0–68.7) of patients. Among patients with fever, 58.1% (95% CI: 37.3–78.8) with a negative test result were treated with an anti-malarial, the proportion being higher in children below five years of age (19/26; 73.1%; 95% CI: 50.3–95.8) than in patients five years and older (17/36; 47.2%; 95% CI: 21.5–73.0).

Table 4 shows the results on the accuracy of routine reading of malaria slides for 158 patients for whom the matching slide result of the expert microscopy was available. The expert slide positivity rate was only 18.4%, significantly lower compared to the routine slide positivity rate (49.4%). Although the sample size was small, in this subset of patients the expert microscopy suggested similar positivity rates between areas of medium-to-high (6/32; 18.8%) and very high transmission areas (23/126; 18.3%). The sensitivity and the specificity of the routine blood slide readings were 58.6% and 52.7%, respectively. The PPV was very low (21.8%) while the NPV was high (85.0%). Although not statistically significant, differences in predictive values were suggested between age groups: the respective PPVs for children below five years and patients five years and older were 45.0% and 13.8%, while the NPVs in the same age groups were 71.4% and 92.3%, respectively (Table 4).

**Table 4:** The slide positivity rate and accuracy of routine malaria microscopy compared to expert microscopy, stratified by age categories

	**Patients <5 years** **n/N,% (95% CI)**	**Patients ≥5 years** **n/N,% (95% CI)**	**All patients** **n/N,% (95% CI)**
Slide positivity rate (routine microscopy)	20/48, 41.7 (25.7–57.5)	58/110, 52.7 (38.8–66.7)	78/158, 49.4 (36.7–62.1)

Slide positivity rate (expert microscopy)	17/48, 35.4 (21.2–49.6)	12/110, 10.9 (4.8–17.0)	29/158, 18.4 (12.5–24.2)

Sensitivity	9/17, 52.9 (24.2–81.7)	8/12, 66.7 (35.6–97.7)	17/29, 58.6 (38.4–78.9)

Specificity	20/31, 64.5 (44.7–84.3)	48/98, 49.0 (34.6–63.3)	68/129, 52.7 (39.2–66.2)

PPV	9/20, 45.0 (18.3–71.7)	8/58, 13.8 (4.7–22.9)	17/78, 21.8 (11.0–32.6)

NPV	20/28, 71.4 (53.5–89.3)	48/52, 92.3 (84.8–99.8)	68/80, 85.0 (78.9–91.1)

## Discussion

These facility-based data stratified by age and transmission intensity reveal a series of important findings directly relevant for the revision of malaria diagnostic recommendations within the context of deployment of new ACT policies in Uganda.

First, the prevalence of malaria parasitaemia among most outpatient groups was lower than initially expected. Indeed, across different transmission settings, which are all traditionally viewed as high malaria transmission intensity areas, only 17–25% of patients above five years of age were parasitaemic. The prevalence rate of 44% was higher in children below five years of age; however, this rate did not exceed 54% even in the areas historically described as having the highest EIRs in the world with between 564 and 1,564 infective bytes per person per year [2] and community-based childhood malaria prevalence rates as high as 79–91% [8]. However, the comparisons between community and facility-based prevalence rates are not straightforward. Intuitively, prevalence rates among sick outpatients can be expected to be at least similar, if not higher, compared to those obtained from household surveys among healthy populations. The self-selection of populations presenting to facilities, who may have higher socio-economic status or different health-seeking behaviour may provide some explanation to this pattern [9,10]. Nevertheless, the findings in this study suggest that in nearly all studied populations, the majority of patients present to outpatient facilities without malaria parasitaemia.

Second, given the low prevalence of malaria (24%), high frequency of fever among outpatients (79%), and health workers' practices largely reflecting recommendations promoting presumptive malaria diagnosis for all febrile patients, the current rates of outpatient malaria over-diagnosis in Uganda are massive, reaching as high as 79% for patients five years and older and remaining high even in the children below five years of age in the study areas of highest malaria transmission (47%). The over-diagnosis rates of routine practices were not much lower than 75% previously reported rates from an area of low and unstable malaria transmission in western Uganda [11].

Yet, it was worrying to observe that despite the minimum overall deviations from national diagnosis recommendations, the current practices in very high transmission areas resulted in a large proportion (49%) of children below five years of age who are not diagnosed although parasitaemic and subsequently not treated for malaria. As observed repeatedly in many studies in the past two decades across Africa [12-16], any deviation from the case definition equalizing fever with malaria increases the specificity of malaria diagnosis, however at the cost of substantially decreased sensitivity. This inevitably results in malaria under-diagnosis, the magnitude of which is dependent on the background prevalence of the disease. The under-diagnosis rates observed in this study present an unacceptable trade-off in children, the population most vulnerable to severe and potentially fatal malaria complications. Therefore, the only potential solution to circumvent problems of malaria misdiagnosis (both over- and under-diagnosis) in Uganda is the change of diagnostic policy from presumptive to parasitological diagnosis of malaria and systematic use of malaria tests for all febrile patients by performing reliable malaria microscopy and introducing rapid diagnostic tests (RDTs).

Finally, a shift from the current presumptive policy based on fever to an effective policy based on malaria diagnostics is unlikely to be trivial. The findings at facilities with functional microscopy in study districts suggest that: 1) febrile patients are not sufficiently tested regardless of whether testing is recommended (46%) or discouraged by guidelines (38%), and 2) there is an overwhelming tendency to ignore negative slide results and prescribe anti-malarial drugs (58%). The underuse of malaria microscopy and the prescription of anti-malarials for negative test results revealed in this study mirror well-described patterns of microscopy use and results interpretation reported in past studies in Kenya [17], Zambia [18,19] and Tanzania [20,21]. With respect to the routine laboratory performance, the quality of routine malaria slide reading was poor (sensitivity and specificity below 60%) and characterized by substantial over-reporting of positive slides across all age groups (55–86%), however, most of the results routinely reported as negative were truly negative, more commonly in patients five years and older (93%) than in children below five years of age (71%). While 29% of false negative results might offer justification for the treatment of test negative children, the findings in patients five years and older are in obvious discordance with prescription practices where nearly all positive tests are routinely treated but also 47% of patients with negative tests. This irrational practice of overruling negative test results, and still performing malaria tests, may have multiple causes but an important one stems out from the decades of ambiguous recommendations translated into pre-service and in-service training programmes and routine practice where malaria microscopy has been seen as a tool to confirm clinical suspicion but rarely as a tool to rule out malaria diagnosis [22,23]. Despite the problems of ensuring large-scale quality of routine microscopy-based malaria case-management across Africa, recent, smaller-scale studies suggest that intensive interventions including at least five days long, integrated in-service training for clinical and laboratory staff supported with supervision and strengthened surveillance may improve some aspects of microscopy-based malaria case-management [24,25]. The challenge remains defining most cost-effective components of such interventions, their routine scale up and maintenance to ensure long-term performance at all facilities providing microscopy services.

The Ugandan Ministry of Health should be commended for the current revisions of malaria diagnostic policies to promote greater access to parasitological-based diagnosis. An important component of this is the introduction of malaria RDTs to complement microscopy specifically in health facilities without laboratory services. Malaria RDTs are accurate under controlled conditions, easy to use and interpret, provide rapid results, and can be performed with the minimum of training and equipment. Yet, the success of this diagnostic strategy encompassing RDTs will be critically dependent on ensuring high accuracy (in particular sensitivity) of RDTs under field conditions [11,26,27], and ensuring a radical change of health workers current practices with emphasis on systematic screening of all fevers, respect of test negative results, and capacity building to manage non-malarial fevers. A quality assurance process at national and peripheral level; development, validation and distribution of clear clinical guidelines to address malaria and non-malaria fevers; health workers', preferably on-job training to introduce tests, initiate translation of guidelines into practice, but also to solve local obstacles to implement microscopy and RDTs; and frequent effective supervision to maintain good practices are the minimum pre-requisites for the success of such a strategy. Therefore, it is important that the introduction of RDTs is accompanied by similar activities to strengthen the quality of microscopy, either as a separate preliminary activity or as an activity integrated within the RDT implementation process [28].

Finally, prior to the national policy change and scale-up, it would be wise that the implementation package introducing RDTs and strengthening malaria microscopy is piloted at district-level in areas of different malaria endemicity. Such pilots should include an operational research component which should monitor routine health workers' practices and real-world accuracy of both diagnostic tools, and evaluate cost and health benefits of a parasitological-based diagnostic strategy in particular compared with the presumptive treatment strategy in the most vulnerable groups such as children below five years of age. The lessons learned from these pilots should help policy makers to improve upon implementation delivery and take informed decisions if all age groups and transmission settings should be the targets of parasitological-based diagnosis for the national scale-up. Failing to approach the introduction of this new diagnostic tool in a careful and evidence-based manner will mean that no lessons have been learned from the experiences of implementing malaria microscopy.

## Conclusion

The traditional view of Uganda as a very high malaria endemic area where most of fevers are due to malaria is unlikely to be true. A large majority of outpatients, across all age groups and transmission areas, with the exception of children below five years of age in very high transmission areas, do not have malaria parasitaemia. National recommendations promoting presumptive malaria diagnosis for all patients with fever and health workers practices following these recommendations result in massive over-diagnosis, particularly in patients five years and older. Equally important, in children below five years of age in very high transmission areas, the current practices result in substantial malaria under-diagnosis. The problem of malaria misdiagnosis can only be tackled by using parasitological-based malaria diagnosis, however, the current experiences of malaria microscopy in Uganda suggest that the potential benefits are not realized because of the poor quality of routine reading of slides and irrational clinical practices. There is need for the change of policy from presumptive to parasitological-based diagnosis, however, the modalities of this change and real-world effectiveness of this strategy will be critically dependent on the quality, and coverage, of the implementation process which must be accompanied by a strong operational research component.

## Competing interests

The authors declare that they have no competing interests.

## Authors' contributions

JN and DZ contributed to study design, data analysis and interpretation of results. JNN contributed to data analysis and interpretation of results. JBR and HC contributed to study design, interpretation of results and policy implications of the findings. RWS and JKT contributed to the conception and design of the study, and interpretation of results. All authors contributed to drafting of the manuscript and all read and approved the final manuscript.
